# Equine neuroborreliosis in Scandinavia: cerebrospinal fluid PCR confirmation and successful treatment in a mare

**DOI:** 10.1186/s13028-026-00879-0

**Published:** 2026-09-01

**Authors:** Sofia Ruiz, Marianne Øverlie, Amanda Bergren, Ingunn Risnes Hellings

**Affiliations:** 1 Dobloug & Ruiz Veterinærtjenester AS, Sankt Mikaels Veg 285, Vang På Hedmarken, 2324 Norway; 2 Bjerke Dyrehospital, Refstadveien 1, Oslo, 0589 Norway; 3 Department of Companion Animal Clinical Sciences, Faculty of Veterinary Medicine and Biosciences, Norwegian University of Life Sciences, Postboks 5003, Ås, 1432 Norway

**Keywords:** Borrelia, Cerebrospinal fluid, Doxycycline, Equine, Neuroinfection

## Abstract

**Background:**

Lyme borreliosis (LB), caused by *Borrelia burgdorferi* sensu lato, is an emerging concern in human medicine, and is a controversial topic in equine medicine. Neuroborreliosis (NB), a neurologic manifestation of LB, remains challenging to diagnose due to nonspecific clinical signs and the lack of definitive diagnostic methods. This report describes the first documented case of equine NB in Scandinavia.

**Case presentation:**

An 11-year-old mare presented with progressive weight loss, ataxia, muscle atrophy, and hyperesthesia. Cerebrospinal fluid (CSF) analysis revealed neutrophilic pleocytosis, elevated protein and a positive PCR result for *Borrelia* spp. The mare showed clinical improvement and long-term recovery following prolonged doxycycline therapy.

**Conclusions:**

This case is the first reported case of neuroborreliosis in a horse in Scandinavia and supports the diagnostic relevance of CSF PCR in conjunction with clinical and cytological findings. It also underscores the potential for recovery in equine NB when early antimicrobial treatment is initiated.

## Background

Lyme borreliosis (LB), caused by members of the *Borrelia burgdorferi* sensu lato (s.l.) complex, is a zoonotic tick-borne disease with growing relevance in both veterinary and human medicine. In the past few decades, climate change and shifting land use have contributed to a gradual expansion in tick habitats, particularly *Ixodes ricinus* in Europe and *I. scapularis* in North America [1, 2]. As a result, LB has become the most commonly reported human tick-borne disease in both regions [2, 3]. However, despite high seroprevalence of *B. burgdorferi* s.l. in horses, there are few cases of documented equine LB [4–14].

While horses can be exposed frequently to *B. burgdorferi* s.l. in endemic areas, which is reflected in the high numbers of seropositive healthy horses [10, 15, 16, 17], clinical disease remains rare, particularly in Europe [4, 8]. Among the various clinical forms, neuroborreliosis (NB) is one of the best documented clinical syndromes attributed to *B. burgdorferi* s.l. infection in horses [4–7, 9, 11, 13], with additional documented manifestations which may include cardiac arrhythmias, uveitis, and cutaneous lesions [10, 18]. Clinical signs of NB are frequently non-specific, and resemble those seen in other musculoskeletal or neurological conditions [10] and may include synovitis, muscle atrophy, progressive weight loss, cranial nerve deficits, laryngeal dysfunction, spinal ataxia, hyperesthesia, fasciculations, cervical and back stiffness [4–7, 9–11, 13].

The diagnosis of equine NB remains controversial due to the lack of a definitive diagnostic standard, the aforementioned high seroprevalence observed in clinically healthy horses, and the variability in the disease’s clinical manifestations. Serological tests are commonly used but cannot distinguish between past and active infection, nor confirm causality of clinical signs [10, 18, 19]. Antigen detection tests on affected tissues may be attempted but are considered low yield, while culture remains technically challenging. Consequently, the sensitivity and specificity of these approaches still require further evaluation [7, 10, 13, 19]. In suspected equine NB cases, uncertainty is compounded by limited data on antimicrobial penetration across a presumed disrupted blood-brain barrier [10]. These factors contribute to delays in diagnosis and treatment, likely impacting clinical outcomes, with a reported median duration of neurologic dysfunction preceding death of approximately 120 days [9, 10].

Here, we describe the first reported case of equine NB in Scandinavia, where positive identification of *Borrelia* spp. by PCR in cerebrospinal (CSF) fluid samples led to a diagnosis, positive response to treatment and long-term survival.

## Case presentation

An 11-year-old Norwegian Warmblood mare was initially examined by a field veterinarian in September 2022 for urticaria and progressive weight loss. Serological allergy testing revealed elevated allergen-specific IgE against *Dermatophagoides farinae* and *Alternaria alternata*, and allergen-specific immunotherapy was initiated. No other relevant pre-existing conditions, concurrent infections, or medications were reported. Over the following week, the mare became increasingly dull, painful on muscle palpation, reluctant to move, and continued to lose weight. She was subsequently referred to Bjerke Dyrehospital (Oslo, Norway).

On admission, the mare was quiet but alert and responsive, with vital parameters within reference limits. She showed marked, bilateral epaxial and cervical muscle atrophy, had a body condition score of 3/9 (Henneke Horse Body Condition Scoring Scale), generalized hyperesthesia, marked cervical pain with reduced range of motion, and lumbar back pain. Neurologic examination revealed grade 1/5 symmetrical ataxia [20] affecting all four limbs, accentuated by head elevation and walking downhill. Both hind limbs displayed mild circumduction, and tail-pull testing indicated right-sided weakness. Reduced distal limb nociception was noted in all four limbs, consistent with diffuse spinal cord involvement. No cranial nerve deficits were detected.

Hematologic findings were consistent with a systemic inflammatory response, characterized by moderate anemia (RBC 5.06 × 10^12/L [6.4–10.4], HCT 22% [30–47], hemoglobin 80 g/L [107–165]), mild leukopenia (3.97 × 10^9/L [4.9–11.1]) and markedly increased serum amyloid A (SAA) (651 mmol/L [< 20]). Serum biochemistry only showed mild hyperglobulinemia (48 g/L [24–47]). Vitamin E levels were within normal limits. Gastroscopy and an oral glucose absorption test were unremarkable. Survey radiographs of the cervical and thoracolumbar spine showed no osseous abnormalities.

CSF collected from the lumbosacral space was slightly cloudy. Laboratory analysis was performed at LABOKLIN GmbH & Co.KG (Bad Kissingen, Germany). Analysis of the CSF revealed a marked increased white blood cell count (2.644 × 10^9 cells/L [0–6]) with neutrophilic pleocytosis and elevated total protein concentration (1.0 g/L [0.05–1.0]), consistent with marked neutrophilic meningoencephalomyelitis. Bacterial culture of the CSF yielded moderate growth of *Staphylococcus capitis*. Polymerase chain reaction testing of CSF was positive for *Borrelia* spp [21]., while PCR for EHV-1, *Streptococcus equi* subsp. *equi* as well as *S. equi* subsp. *zooepidemicus* were all negative. ELISA testing for antibodies against tick-borne encephalitis virus was negative.

Based on the compatible clinical presentation, exclusion of alternative infectious etiologies, and CSF cytologic and molecular findings, a diagnosis of neuroborreliosis was made. Treatment was initiated with doxycycline (10 mg/kg PO q12h (Karidox 100 mg/ml, Alivira, Barcelona, Spain) and flunixin meglumine (1.1 mg/kg IV q24h (Flunixin Biovet Vet 50 mg/ml, ScanVet, Fredensborg, Denmark).

Within 7 days of initiating treatment, the mare showed marked improvement in mentation and comfort, accompanied by normalization of hematologic parameters and SAA (381 mmol/L). NSAIDs were discontinued after 10 days, but clinical deterioration followed. Meloxicam (0.6 mg/kg PO q24h (Metacam 15 mg/ml, Boehringer Ingelheim Vetmedica GmbH, Ingelheim, Germany) was then introduced for an additional five days, leading to clinical stabilization. Inflammatory markers, including SAA, returned to within reference limits at 13 days after treatment initiation, and the mare was discharged after 19 days of hospitalization with continued doxycycline.

At re-examination 10 days post-discharge, the mare was brighter, had gained 18 kg, but showed mild residual neurologic deficits (grade 1 ataxia, hind limb toe dragging, and right-sided weakness). SAA was normal (4mmol/L); hematology showed mild anemia (RBC 6.15 × 10^12/L [6.4–10.4], HCT 26,7% [30–47] and HGB 97 g/L [107–165]) and leukopenia (4.71 × 10^9/L [4.9–11.1])). CSF was clear, with near-normal cytologic findings (3 × 10^6 cells/µL [0–6]), protein 1.07 g/L [0.05–1.0]), negative bacterial culture, and negative PCR for *Borrelia* spp., supporting treatment efficacy. Antimicrobial therapy was continued for a total of 7 weeks.

The mare continued to improve, with progressive weight gain and normalization of behavior. After two months of rest, ridden exercise was reintroduced without recurrence of neurologic or musculoskeletal abnormalities. At owner follow-up in early 2026, approximately 3.5 years after the initial presentation, no recurrence had been reported; the mare remained clinically healthy and was participating in recreational dressage training.

### Discussion and conclusions

This is the first case of neuroborreliosis reported from a horse in Norway and from Scandinavia. The case had many of the typical clinical signs previously reported in horses with neuroborreliosis, but none of these signs are pathognomonic. In human medicine, criteria suggested for making a diagnosis of LD include known or presumed tick exposure, compatible clinical signs, exclusion of other conditions, and supportive laboratory findings.

The mare in this report had a relevant exposure history, having been pastured in southeastern Norway, where the prevalence of *I. ricinus* and *B. burgdorfer* s.l. is well documented [1, 22]. Supporting the likelihood of exposure, studies investigating the presence of antibodies against *B. burgdorferi* s.l. in Scandinavian equine populations reported an apparent seroprevalence of 16.8% in Sweden [15], 29.0% in Denmark [16] and 47% in Norway [17].

This case presented with signs commonly associated with equine NB, including weight loss, muscle atrophy, proprioceptive and spinal ataxia, hyperesthesia and cervical stiffness. Although other signs such as cranial nerve deficits, laryngeal dysfunction, synovitis and fasciculations were not observed, previous reports emphasize that equine Lyme disease presents variably and lacks a pathognomonic pattern [9, 10].

Laboratory support included a CSF cytological evaluation showing xanthochromia, neutrophilic pleocytosis, and elevated total protein – findings consistent with previous equine neuroborreliosis cases [7, 9, 13]. The presence of *S. capitis* in the bacterial culture of the fluid, a commensal organism often found in human skin, was deemed the result of inadvertent sample contamination. Although it has been rarely associated with central nervous system infections, such as meningitis in immunocompromised adult human patients [23], its role as a primary pathogen in equine neurology remains undocumented to the author’s knowledge. Lastly, the PCR analysis of the CSF sample yielded a positive result for *Borrelia* spp. DNA, further supporting the diagnosis of neuroborreliosis in this horse. As the PCR assay identified *Borrelia* spp. without species-level characterization, the infecting genospecies could not be determined. Therefore, it remains unknown whether the infection was caused by *B. burgdorferi* sensu stricto, *B. garinii*, *B. afzelii*, or another member of the *B. burgdorferi* s.l. complex. While PCR of CSF is considered a low-yield procedure due to limited organism presence in fluid [5], positive results are regarded as clinically meaningful, particularly in the absence of findings suggesting involvement of other pathogens and when serology is unavailable, as in this case. Notably, antemortem diagnoses of equine NB have previously been reported based on a combination of supportive findings, including a positive CSF PCR [7, 13]. However, the conventionally available PCR methods may not always be sufficient, and newer techniques such as the genomic hybrid capture may have improved sensitivity for detecting *B. burgdorferi* DNA in CSF samples [13].

Antibody testing was not performed in this case, which limits full diagnostic certainty. However, growing evidence suggests that serology has limited diagnostic utility in equine NB, as several reported cases have yielded equivocal or negative results in serum and/or CSF samples [5–7, 9, 13, 19]. Consequently, serology remains a supportive rather than definitive tool, and its omission in this case does not preclude a justified diagnosis.

Another consideration in the diagnostic process involved the possibility of viral encephalitis, including EHV-1, borna disease and flaviviral infections such as West Nile virus (WNV), Usutu virus (USUV), tick-borne encephalitis virus (TBEV) and Louping ill virus (LIV), as part of the differentials in horses with neurologic signs such as the one presented in this case. Serological testing for TBEV was performed and was negative, making acute TBEV infection unlikely. However, WNV, USUV and LIV were not specifically investigated, representing a limitation of this report. At the time (2022), WNV had only been reported in equine cases in central and southern Europe, and the majority of infections are either subclinical or result in mild clinical signs [24]. Furthermore, the mare had no history of travelling outside Norway and WNV has so far not been reported in horses in Norway. Equine USUV- related clinical signs have not been described to date. LIV, an extremely rare cause of encephalitis in horses, shares *I. ricinus* as a vector with both TBEV and Borrelia, but reported cases are mostly confined to enzootic areas of the British Isles [24]. Nevertheless, the positive PCR on CSF for *Borrelia* spp., the neutrophilic pleocytosis identified in the CSF, the marked clinical improvement following doxycycline treatment, and normalization of inflammatory markers strongly support neuroborreliosis as the most likely diagnosis.

Long-term recovery was achieved following treatment with oral doxycycline in combination with NSAIDs, which is an infrequently reported outcome based on previous cases of equine NB, many of which deteriorated despite treatment [4, 5, 8, 9]. Although doxycycline is seen to have low CSF penetration in healthy horses [25], its efficacy in this case may reflect improved access during inflammatory stages and early therapeutic intervention. Moreover, the successful antibiotic efficacy seen was later supported by the absence of detectable *Borrelia* DNA in the follow-up CSF sample, which aligns to the favorable outcome previously reported in a comparable equine NB case treated with doxycycline as the sole antimicrobial agent [7].

In conclusion, this case expands the documented geographic range of equine neuroborreliosis by presenting the first report from Scandinavia. It also provides molecular confirmation of *Borrelia* spp. DNA in cerebrospinal fluid and adds to the limited number of cases with documented treatment success and sustained long-term recovery. This highlights the potential for horses to recover from NB when appropriate and timely antimicrobial therapy is initiated. Consequently, research on diagnostic and therapeutic methods, as well as further epidemiological evaluations and insights made on a case-by-case report basis, may allow progress in the management of neuroborreliosis in the living horse.

## Data Availability

No datasets were generated or analysed during the current study.

## Declarations

### Prior publication

Data have not been published previously.

## Abbreviations

AAEP: American Association of Equine Practitioners
CSF: Cerebrospinal fluid
LB: Lyme borreliosis
NB: Neuroborreliosis
PCR: Polymerase chain reaction
SAA: Serum amyloid A
